# Impact of Cadmium Exposure on the Association between Lipopolysaccharide and Metabolic Syndrome

**DOI:** 10.3390/ijerph120911396

**Published:** 2015-09-11

**Authors:** Seung Jin Han, Kyoung Hwa Ha, Ja Young Jeon, Hae Jin Kim, Kwan Woo Lee, Dae Jung Kim

**Affiliations:** 1 Department of Endocrinology and Metabolism, Ajou University School of Medicine, 164, World Cup-ro, Yeongtong-gu, Suwon 443-380, Korea; E-Mails: hsj@ajou.ac.kr (S.J.H.); khha84@gmail.com (K.H.H.); twinstwins@hanmail.net (J.Y.J.); jinkim@ajou.ac.kr (H.J.K.); lkw65@ajou.ac.kr (K.W.L.); 2 Cardiovascular and Metabolic Disease Etiology Research Center, Ajou University School of Medicine, 164, World Cup-ro, Yeongtong-gu, Suwon 443-380, Korea

**Keywords:** microbes, metabolic syndrome, cadmium, lipopolysaccharide

## Abstract

Cadmium (Cd) is an environmental contaminant that has a direct impact on the gut microbiome. Perturbations in the gut microbiome have been linked to metabolic disorders associated with inflammation generated by lipopolysaccharide (LPS). We investigated the impact of Cd on the association between LPS and metabolic syndrome. The study population consisted of 200 apparently healthy subjects (30–64 years of age; 96 men, 104 women). Serum LPS and blood Cd concentrations were measured by ELISA and graphite furnace-atomic absorption spectrophotometry (GF-AAS), respectively. The highest LPS quartile was associated with a greater prevalence of metabolic syndrome in men. There was a significant association between LPS activity and metabolic syndrome in men with blood Cd concentrations higher than the 50th percentile (OR = 3.05, 95% CI = 1.39–6.70); however, this relationship was not significant in men with blood Cd concentrations lower than the 50th percentile. The results of this study provide evidence for a strong association between high LPS activity and the prevalence of metabolic syndrome in men with relatively high blood Cd concentrations. Therefore, exposure to Cd may potentiate the association between LPS and metabolic syndrome in men.

## 1. Introduction

Metabolic syndrome describes a group of risk factors, including central obesity, hyperglycemia, dyslipidemia and hypertension, that is associated with the development of diabetes and cardiovascular disease [1,2]. These metabolic disorders are associated with low-grade inflammation, which is a significant contributor to the development of these diseases [3,4]. The prevalence of metabolic syndrome has been increasing steadily over the last few decades. According to the Korean National Health and Nutrition Examination Survey (KNHANES), the age-adjusted metabolic syndrome prevalence in Korea has increased from 24.9%–31.3% from 1998–2007 [5].

Previous research has mostly focused on the roles that over-nutrition and physical inactivity have played in the development of metabolic syndrome; however, new factors, such as the intestinal microbiome and environmental chemicals, have been the subject of more recent research [6]. Recent studies have found mounting evidence that gut microbes play a critical role in whole-body metabolism by affecting energy homeostasis, glucose metabolism and low-grade inflammation associated with metabolic syndrome [3,7,8,9]. It has been suggested that the gut microbiome is a source of lipopolysaccharide (LPS), a major component of the cell wall in Gram-negative bacteria that triggers inflammation [10]. Cani *et al.* (2007) have shown that gut bacteria can initiate the inflammatory state of obesity and insulin resistance through the activity of LPS, which can trigger the inflammatory process by binding to the CD14-toll-like receptor 4 (TLR-4) complex on the surface of innate cells. Most circulating endotoxin is bound to lipoproteins, and HDL cholesterol is the main acceptor involved in the sequestration of LPS from the circulation under physiological conditions [11]. Under conditions of acute infection and inflammation, LPS is redistributed toward LDL and VLDL lipoproteins [12]. Previous studies have shown that a high LPS/HDL ratio is associated with cardiovascular disease and metabolic syndrome [11,13].

Cadmium (Cd) is a toxic heavy metal with a long half-life (10–30 years) in humans. Exposure can occur through contaminated food, water or air. Cd has been linked to an increased risk of renal, cardiovascular, neurologic and developmental diseases and, thus, mortality in humans [14]. The gastrointestinal tract is a key organ involved in processing xenobiotics, and gut microbes likely play an important role in the bioavailability and toxicity of heavy metals. Recent animal studies found that exposing mice to Cd led to a profound toxic effect on the microbiome in the intestinal tract [15,16,17]. Furthermore, Liu *et al.* (2014) found that oral exposure to Cd induced gut barrier impairment and altered the diversity, as well as the total number of microbial species present in the intestinal tracts of mice.

To our knowledge, no previous studies have explored the impact of Cd exposure on LPS production and metabolic syndrome prevalence. Therefore, we investigated whether Cd exposure affects the association between bacterial endotoxin and metabolic syndrome in humans.

## 2. Methods

### 2.1. Subjects

This research is part of an ongoing, population-based study in Korean adults (30–64 years of age) conducted at the Cardiovascular and Metabolic Diseases Etiology Research Center (CMERC). Research at CMERC began in 2013 in an effort to improve cardiovascular and metabolic disease predictive models, to discover new risk factors and biomarkers, to explore new prevention strategies and to gather direct evidence relevant to the prevention of cardiovascular and metabolic diseases.

This study was conducted at CMERC in 200 healthy volunteers (30–64 years of age; 96 men, 104 women) from the cities of Suwon, Yongin and Hwasung, Republic of Korea. Any volunteers who had been diagnosed with malignant tumors within the previous two years, who were currently receiving medical treatment or who had a history of myocardial infarction, stroke or other cardiovascular disease were excluded from the study. In addition, anyone with acute illness, current evidence of acute or chronic inflammatory or infective disease, recent surgery, renal disease or hepatic disease was also excluded. All subjects provided written informed consent, and in accordance with the Declaration of Helsinki of the World Medical Association, the Ajou University Institutional Review Board approved this study protocol (IRB No. AJIRB-BMR-SUR-13-272). The details of the study design and procedures have been described previously [18].

### 2.2. Anthropometric and Laboratory Measurements

Demographic data were collected from study participants by trained interviewers, which included the following: age, gender, cigarette smoking status (never, former, current), alcohol consumption (grams/day) and physical exercise (low, moderate, high). Physical exercise was measured using the international physical activity questionnaire short form instrument, and the data were divided into three categories based on standard scoring criteria (http://www.ipaq.ki.se).

An automatic height-weight scale (BSM330; InBody Co., Ltd., Seoul, Korea) was used to measure height (cm) and weight (kg) to a resolution of 0.1 cm and 0.1 kg, respectively. Body mass index (BMI) was calculated as weight/height^2^ (kg/m^2^). Waist circumference was measured using a measuring tape (Seca GmbH, Hamburg, Germany) at the midpoint between the bottom edge of the last rib and the high point of the iliac crest to the nearest 0.1 cm with the subject in an upright position.

Blood pressure (BP) was measured after 5 min of rest in the sitting position. Three measurements were taken in the right arm using an electronic manometer (HEM-7080IC, Omron Healthcare Co., Ltd., Kyoto, Japan), and the average of the second and third measurements was used in subsequent analyses. Blood samples were obtained after a fasting period of at least 8 h. Total cholesterol, triglyceride (TG), and HDL cholesterol levels were quantified using an enzyme method, and fasting serum glucose levels were measured using a colorimetric method. Serum LPS levels were measured using a competitive inhibition enzyme immunoassay technique (Kamiya Biomedical Co., Seattle, WA, USA). Since HDL cholesterol is the main factor involved in the sequestration of circulating LPS, we used the LPS/HDL ratio as a functional measure of LPS activity [11,12,13,19]. Blood Cd concentrations were measured by graphite furnace-atomic absorption spectrophotometry with Zeeman background correction (AAnalyst TM 800, Perkin Elmer, Singapore, Singapore).

### 2.3. Metabolic Syndrome Criteria

Metabolic syndrome was defined according to the criteria established by the National Cholesterol Education Program Adult Treatment Panel (NCEP/ATP III), using the adjusted waist circumference for Koreans [20]. Accordingly, participants with three or more of the following five criteria were defined as having metabolic syndrome: (i) abdominal obesity according to waist circumference (defined as Korean-specific waist circumference cutoff values of ≥90 cm for men and ≥85 cm for women); (ii) systolic blood pressure ≥130 mmHg or diastolic blood pressure ≥85 mmHg or on antihypertensive medication; (iii) elevated fasting blood glucose (≥100 mg/dL); (iv) hypertriglyceridemia (≥150 mg/dL); and (v) low serum HDL cholesterol (<40 mg/dL in men and <50 mg/dL in women).

### 2.4. Statistical Analyses

Normally-distributed variables are presented as the means ± standard deviation (SD), whereas skewed variables are presented as medians with the interquartile range (IQR). Statistical differences between groups were determined using the Student’s *t*-test or the Mann–Whitney U-test and chi-square (χ^2^) test, when appropriate. LPS and LPS/HDL ratios and skewed variables were log transformed. Odds ratios (OR) and 95% confidence intervals (CI) for predicting metabolic syndrome based on the LPS or LPS/HDL ratio were obtained from logistic regression models after controlling for potential covariates. All statistical analyses were conducted using SAS, Version 9.2 (SAS Institute, Cary, NC, USA). Results were considered statistically significant at a *p*-value <0.05.

## 3. Results

Demographic data about the study population, categorized by sex and metabolic syndrome status, are presented in Table 1. In our study population, metabolic syndrome was more prevalent in men than women (30.2% *vs.* 16.4%; *p* = 0.031). Subjects with metabolic syndrome had a higher BMI, waist circumference, BP, fasting glucose level and TG level and lower HDL cholesterol compared to those without metabolic syndrome.

**Table 1:** Demographic data of the study population categorized by sex and metabolic syndrome status.

	Men					Women				
	No Metabolic Syndrome		Metabolic Syndrome		*p*	No Metabolic Syndrome		Metabolic Syndrome		*p*
N (%)	67	(69.8)	29	(30.2)		87	(83.7)	17	(16.4)	
Age, years	48.4	(9.1)	50.3	(7.6)	0.321	47.8	(49.0)	52.0	(7.0)	0.042
Current smoking, n (%)	24	(35.8)	9	(31.0)	0.826	2	(2.3)	0	(0.0)	1.000
Alcohol consumption, g/day	10.9	(30.2)	9.4	(36.5)	0.825 ^a^	0.0	(1.3)	0.0	(1.4)	0.798 ^a^
Physical exercise, n (%)										
Low	29	(43.3)	12	(41.4)	0.949	45	(51.7)	6	(35.3)	0.314
Moderate	26	(38.8)	11	(37.9)		38	(43.7)	9	(52.9)	
High	12	(17.9)	6	(20.7)		4	(4.6)	2	(11.8)	
BMI, kg/m^2^	24.8	(2.1)	27.8	(2.6)	<0.001	23.1	(2.6)	26.0	(2.4)	<0.001
Waist circumference, cm	85.2	(6.0)	93.5	(5.5)	<0.001	75.0	(6.5)	84.4	(6.3)	<0.001
Systolic BP, mmHg	121.0	(10.1)	126.5	(11.2)	0.020	112.2	(15.1)	124.1	(19.7)	0.006
Diastolic BP, mmHg	78.5	(8.3)	82.4	(7.7)	0.035	71.0	(10.8)	78.0	(13.3)	0.021
Fasting glucose, mg/dL	89.5	(8.8)	101.3	(22.8)	0.011	84.7	(7.1)	103.7	(24.7)	0.006
HDL cholesterol, mg/dL	47.0	(10.4)	41.4	(10.0)	0.016	54.7	(10.8)	40.0	(6.7)	<0.001
Triglycerides, mg/dL	166.0	(179.8)	208.0	(92.0)	0.001 ^a^	86.0	(64.0)	191.0	(80.0)	<0.001 ^a^
LPS, ng/mL	36.8	(52.2)	69.2	(152.8)	0.122 ^a^	70.5	(82.3)	35.6	(42.6)	0.065 ^a^
LPS/HDL ratio, units	0.8	(1.3)	1.7	(5.7)	0.122 ^a^	1.3	(1.5)	1.0	(1.0)	0.792 ^a^
Cd, μg/L	1.0	(0.3)	1.1	(0.4)	0.122	1.4	(0.7)	1.5	(0.4)	0.464
Metabolic syndrome components, n (%)										
High waist circumference	12	(17.9)	25	(86.2)	<0.001	6	(6.9)	10	(58.8)	<0.001
Low HDL cholesterol	10	(14.9)	15	(51.7)	<0.001	25	(28.7)	17	(100.0)	<0.001
High TG	21	(31.3)	22	(75.9)	<0.001	9	(10.3)	13	(76.5)	<0.001
High BP	24	(35.8)	22	(75.9)	0.001	18	(20.7)	9	(52.9)	0.013
High glucose	7	(10.5)	15	(51.7)	<0.001	1	(1.2)	9	(52.9)	<0.001

Data are presented as the means ± standard deviation (SD), N (%), or medians (interquartile ranges). BMI, body mass index; BP, blood pressure; TG, triglyceride; LPS, lipopolysaccharide; Cd, cadmium. *p*-values represent the difference between the presence and absence of metabolic syndrome for each variable within the same sex using the *t*-test, χ2 test, or the Mann–Whitney U-test (*p* ^a^ value), when appropriate.

The associations between metabolic syndrome and risk factors, according to the LPS, are presented in Table 2 and Table 3. The ORs and 95% CIs for metabolic syndrome and associated risk factors were calculated based on quartiles of log-transformed serum LPS following covariate adjustment. The covariate for Model 1 was age, and the covariates for Model 2 were age, smoking status, alcohol consumption and physical exercise. The highest LPS quartile was associated with a greater prevalence of metabolic syndrome among men (Model 1: OR = 4.32; 95% CI = 1.17–15.90; Model 2: OR = 4.69; 95% CI = 1.23–17.94; Table 2). Among the five components of metabolic syndrome, the ORs for low HDL cholesterol and hypertension were significantly associated with the LPS/HDL ratio in men. These results did not change when subjects were divided into quartiles by the LPS/HDL ratio instead of LPS (Model 1: OR = 3.70; 95% CI = 1.00–13.64; Model 2: OR = 3.85; 95% CI = 1.03–14.40; Supplementary Table S1). Even though, the highest LPS quartile was associated with a lower prevalence of metabolic syndrome among women (Model 1: OR = 0.14; 95% CI = 0.03–0.81; Model 2: OR = 0.15; 95% CI = 0.03–0.87; Table 3). There were no significant associations between the LPS/HDL ratio and metabolic syndrome in women according to the quartiles of the LPS/HDL ratio (Supplementary Table S2).

Next, we analyzed the association between the LPS and metabolic syndrome based on blood Cd concentrations (Table 4). There was a significant association between LPS and metabolic syndrome in men with blood Cd concentrations higher than the 50th percentile (OR = 3.05; 95% CI = 1.39–6.70). However, this relationship was not significant in men with blood Cd concentrations lower than the 50th percentile. There was no significant association between the LPS and metabolic syndrome, based on blood Cd concentrations, among the women in our study. These results were consistent when subjects were divided into quartiles by the LPS/HDL ratio (Supplementary Table S3).

## 4. Discussion

To the best of our knowledge, this is the first study to investigate the effect of Cd exposure on the association between the bacterial endotoxin LPS and metabolic syndrome. The results of this study indicate that higher LPS activity is strongly associated with metabolic syndrome in men, and this association was statistically significant in men with relatively high blood Cd concentrations.

The human microbiome consists of approximately 100 trillion (10^14^) bacteria, weighing a total of 1–2 kg, an amount that is at least 10-fold greater than the cells that make up the human body [21,22]. Therefore, it is widely acknowledged that this microbial consortium provides pivotal metabolic and biological functions that cannot be performed by human metabolic cells alone [23]. Gut microbiota-derived LPS is involved in the onset and progression of inflammatory and metabolic diseases. Gut microbes also mediate the absorption, distribution, metabolism and excretion of environmental pollutants, such as heavy metals [24]. Therefore, the effect of metal toxicity on microbiota has been the focus of recent studies; however, the effect of chronic low-dose exposure of heavy metals on metabolic endotoxemia is still not well understood.

**Table 2:** Odds ratios (OR) and 95% confidence intervals (CI) for metabolic syndrome and associated risk factors according to the LPS in men.

	LPS Quartiles						
	Quartile 1 (<26.7)	Quartile 2 (26.7–43.4)		Quartile 3 (43.4–1028)		Quartile 4 (≥102.8)	
	OR	OR	(95% CI)	OR	(95% CI)	OR	(95% CI)
Metabolic syndrome							
Model 1	1.00	1.19	(0.30–4.65)	1.14	(0.29–4.49)	4.32	(1.17–15.90)
Model 2	1.00	1.18	(0.30–4.63)	1.17	(0.30–4.66)	4.69	(1.23–17.94)
High waist circumference							
Model 1	1.00	0.94	(0.27–3.31)	1.89	(0.57–6.33)	2.70	(0.80–9.09)
Model 2	1.00	0.98	(0.27–3.54)	2.17	(0.62–7.58)	3.14	(0.86–11.48)
Low HDL cholesterol							
Model 1	1.00	3.06	(0.68–13.79)	3.13	(0.69–14.21)	3.29	(0.74–14.58)
Model 2	1.00	3.77	(0.76–18.62)	4.36	(0.85–22.47)	5.43	(1.04–28.50)
High TG							
Model 1	1.00	1.79	(0.56–5.71)	0.77	(0.23–2.53)	1.29	(0.40–4.11)
Model 2	1.00	1.95	(0.60–6.37)	0.82	(0.24–2.77)	1.43	(0.43–4.80)
High BP							
Model 1	1.00	1.28	(0.39–4.22)	1.00	(0.30–3.33)	4.47	(1.25–16.00)
Model 2	1.00	1.27	(0.37–4.42)	0.90	(0.25–3.24)	4.99	(1.28–19.38)
High blood glucose							
Model 1	1.00	4.34	(0.74–25.59)	4.83	(0.83–28.04)	3.77	(0.59–24.02)
Model 2	1.00	4.27	(0.72–25.42)	4.45	(0.74–26.71)	3.58	(0.53–24.34)

Model 1: adjusted for age; Model 2: Model 1 plus additional adjustments for smoking, alcohol consumption and physical exercise; BP, blood pressure; TG, triglyceride.

**Table 3:** Odds ratios (OR) and 95% confidence intervals (CI) for metabolic syndrome and associated risk factors according to the LPS in women.

	LPS Quartiles						
	Quartile 1 (<34.2)	Quartile 2 (34.2–63.0)		Quartile 3 (63.0–112.1)		Quartile 4 (≥112.1)	
	OR	OR	(95% CI)	OR	(95% CI)	OR	(95% CI)
Metabolic syndrome							
Model 1	1.00	0.32	(0.08–1.32)	0.30	(0.07–1.35)	0.14	(0.03–0.81)
Model 2	1.00	0.31	(0.08–1.31)	0.30	(0.07–1.38)	0.15	(0.03–0.87)
High waist circumference							
Model 1	1.00	0.77	(0.20–2.95)	0.44	(0.10–2.01)	0.27	(0.05–1.49)
Model 2	1.00	0.77	(0.20–2.98)	0.44	(0.10–2.01)	0.27	(0.05–1.50)
Low HDL cholesterol							
Model 1	1.00	0.36	(0.11–1.21)	0.64	(0.21–2.02)	1.22	(0.40–3.73)
Model 2	1.00	0.37	(0.11–1.24)	0.63	(0.20–1.99)	1.17	(0.38–3.00)
High TG							
Model 1	1.00	0.31	(0.08–1.29)	0.42	(0.10–1.72)	0.53	(0.14–1.98)
Model 2	1.00	0.32	(0.08–1.33)	0.41	(0.10–1.68)	0.49	(0.13–1.80)
High BP							
Model 1	1.00	0.82	(0.21–3.17)	0.65	(0.15–2.79)	2.20	(0.63–7.75)
Model 2	1.00	0.89	(0.22–3.55)	0.59	(0.13–2.64)	1.91	(0.52–6.97)
High blood glucose							
Model 1	1.00	0.39	(0.06–2.42)	0.77	(0.15–3.99)	0.19	(0.02–1.85)
Model 2	1.00	0.40	(0.06–2.59)	0.87	(0.16–4.80)	0.23	(0.02–2.38)

Model 1: adjusted for age; Model 2: Model 1 plus additional adjustments for alcohol consumption and physical exercise; BP, blood pressure; TG, triglyceride.

**Table 4:** The association between the LPS and metabolic syndrome based on blood Cd concentrations.

	Men				Women			
	N (%)		OR	(95% CI)	N (%)		OR	(95% CI)
Metabolic syndrome								
Cd < 50th percentile	11	(22.9)	1.00	(0.44–2.29)	6	(11.5)	1.06	(0.42–2.00)
Cd ≥ 50th percentile	18	(37.5)	3.05	(1.39–6.70)	11	(21.2)	0.52	(0.23–1.19)
High waist circumference								
Cd < 50th percentile	17	(35.4)	1.37	(0.66–2.83)	8	(15.4)	1.52	(0.63–3.71)
Cd ≥ 50th percentile	20	(41.7)	1.57	(0.83–2.95)	8	(15.4)	0.46	(0.18–1.19)
Low HDL cholesterol								
Cd < 50th percentile	8	(16.7)	0.98	(0.37–2.60)	18	(34.6)	1.50	(0.79–2.87)
Cd ≥ 50th percentile	17	(35.4)	2.11	(0.97–4.60)	24	(46.2)	1.05	(0.59–1.87)
High TG								
Cd < 50th percentile	20	(41.7)	0.98	(0.49–1.95)	7	(13.5)	1.08	(0.46–2.56)
Cd ≥ 50th percentile	23	(47.9)	1.50	(0.82–2.77)	15	(28.9)	0.96	(0.51–1.79)
High BP								
Cd < 50th percentile	18	(37.5)	1.81	(0.85–3.85)	12	(23.1)	1.71	(0.78–3.73)
Cd ≥ 50th percentile	28	(58.3)	1.62	(0.79–3.32)	15	(28.9)	1.16	(0.61–2.18)
High blood glucose								
Cd < 50th percentile	10	(20.8)	1.08	(0.45–2.57)	4	(7.7)	1.27	(0.38–4.26)
Cd ≥ 50th percentile	12	(25.0)	1.67	(0.75–3.70)	6	(11.5)	0.91	(0.34–2.46)

Model adjusted for age, smoking, alcohol consumption and physical exercise in men and age, alcohol consumption and physical exercise in women. Cd, cadmium; BP, blood pressure; TG, triglyceride. The cutoff values for the 50th percentile of Cd are 0.96 μg/L for men and 1.28 μg/L for women.

Cd is a toxic heavy metal and environmental pollutant used in paint, electroplating, batteries and fertilizers [17]. Humans are exposed through contaminated food, water and air. While extremely small amounts of Cd (only ~0.001% of total body burden) are excreted daily in urine [25], there is no biochemical mechanism that can effectively eliminate Cd from the body, so it accumulates over time [26]. Previous studies have documented the association between Cd exposure and chronic diseases; even at low concentrations, Cd exposure has been linked to pre- and type 2 diabetes, hypertension and cardiovascular disease, all of which contribute to increased mortality [27,28,29,30].

Up until now, the studies that have investigated the interaction between Cd and LPS have only been conducted in animal models. Of those studies, exposure to an ostensibly ineffectual dose of Cd was associated with the promotion of LPS-induced oxidative stress and liver damage in rats [31]. Satarug *et al.* found that Cd exposure plus LPS led to synergistic renal toxicity in rats [32]. Our study found a strong interaction between LPS and metabolic syndrome among high blood Cd concentrations in humans.

Previous animal studies on the association between Cd and gut and the association between LPS and metabolic syndrome have provided some partial insight for us to build up our own potential mechanisms involved in the effects of Cd on LPS activity and metabolic syndrome. One mechanism is that Cd may increase gut permeability, thereby potentially increasing the absorption of the LPS endotoxin, resulting in endotoxemia [33]. In mice, Cd exposure led to decreased thickness of the intestinal mucus layer, supporting the mechanism of increased gut permeability [15]. A second mechanism is that Cd can reduce the growth and abundance of total intestinal bacteria, as well as reduce the ratio of Bacteroidetes/Firmicutes, which has been demonstrated in mice [15,17] and has been associated with obesity, both in mice and humans [34,35,36]. It has been suggested that alterations in the gut microbiome, including composition and diversity, could result in increased gut permeability via alterations in the function of tight-junction proteins [33,34]. A third mechanism is that Cd could alter the metabolism of short-chain fatty acids (SCFAs), which are produced through the fermentation of dietary fiber by intestinal bacteria. Liu *et al.* found that Cd exposure led to a decrease in the expression of genes involved in butyrate and acetate synthesis, as well as a decrease in the levels of SCFAs in the colon of mice [15]. SCFAs like butyrate play an important role in energy metabolism. Butyrate provides energy for colonic epithelial cells and maintains intestinal integrity. This may contribute to the prevention of endotoxemia, a process resulting from the translocation of LPS [37]. In humans, there were fewer butyrate-producing bacteria in the gut microbiome of patients with type 2 diabetes compared to healthy subjects [38,39]. Furthermore, butyrate has been shown to improve insulin sensitivity and to increase energy expenditure by enhancing mitochondrial activity in a mouse model [40]. Based on these studies, we hypothesized that our subjects with relatively low exposure of Cd may be less prone to damage of the intestinal tract and reduced growth of microbiota, the change of the ratio of Bacteroidetes/Firmicutes and metabolism of SCFAs. As with other epidemiologic studies on environmental exposures, however, it is inherently difficulty to isolate the effects solely related to Cd. Further research is still needed to elucidate the mechanisms involved in the toxic effects of Cd exposure on LPS activity and metabolic syndrome.

In our study population, there are sex differences in the association between LPS and metabolic syndrome. There are some studies that have found evidence of sex-related differences in the inflammation process. Gnauck *et al.* [41] also found no correlation between body fat content or BMI and levels of LPS in healthy women. LPS-stimulated blood cells produced higher levels of cytokines in males compared to females [42]. In another study, male astrocytes showed enhanced expression of IL-6, TNF-α and IL-1β in response to LPS, compared to female astrocytes [43]. Therefore, differences between men and women in the levels of body fat and the inflammatory response to LPS may have contributed to our results. However, further studies are needed to confirm this suggestion and to determine the exact mechanism involved.

We should note that there are several limitations to interpreting the results of our study. First, the cross-sectional design did not allow us to draw causal inferences from the observed relationships. Second, we assessed exposure to environmental Cd by measuring blood Cd concentrations. Blood Cd concentrations are usually indicative of recent Cd exposure, but also correlate well with total body Cd load and urine Cd, which is a biomarker of lifetime Cd exposure [44,45]. Third, our findings could also be limited by difficulties in fully accounting for potential confounding variables, including diet, socioeconomic status or other environmental factors.

## 5. Conclusions

The results of this study provide evidence for a strong association between high LPS activity and the prevalence of metabolic syndrome in men with relatively high blood Cd concentrations. Given the widespread exposure to Cd and the increasing worldwide burden of metabolic syndrome, these data have substantial public health implications for the general population. Further studies are needed to confirm this association and to investigate the mechanisms involved in the effects of Cd on LPS and metabolic syndrome.

## Supplementary Files

Supplementary File 1
