# Supplementary material for: Impact of Cadmium Exposure on the Association between Lipopolysaccharide and Metabolic Syndrome
Source: Int J Environ Res Public Health. 2015 Sep 11;12(9):11396–409. doi: 10.3390/ijerph120911396 (PMC4586682; doi:10.3390/ijerph120911396)
Supplement: Supplementary File 1 [file ijerph-12-11396-s001.pdf]

Impact of Cadmium Exposure on the Association between Lipopolysaccharide and Metabolic Syndrome

Table S1. Odds ratios (OR) and 95% confidence intervals (CI) for metabolic syndrome and associated risk factors according to the LPS/HDL ratio in men.

	LPS/HDL Quartiles						
	Quartile 1 (<0.6)	Quartile 2 (0.6–1.1)		Quartile 3 (1.1–2.1)		Quartile 4 (≥2.1)	
	OR	OR	(95% CI)	OR	(95% CI)	OR	(95% CI)
Metabolic syndrome							
Model 1	1.00	1.32	(0.34 – 5.16)	1.56	(0.41 – 5.92)	3.70	(1.00 – 13.64)
Model 2	1.00	1.28	(0.32 – 5.15)	1.56	(0.41 – 5.97)	3.85	(1.03 – 14.40)
High waist circumference							
Model 1	1.00	1.04	(0.30 – 3.65)	2.45	(0.74 – 8.13)	2.34	(0.69 – 7.93)
Model 2	1.00	0.95	(0.26 – 3.53)	3.05	(0.86 – 10.82)	2.37	(0.67 – 8.45)
Low HDL cholesterol							
Model 1	1.00	7.55	(0.83 – 68.55)	9.56	(1.07 – 85.28)	18.42	(2.12 – 160.20)
Model 2	1.00	5.10	(0.52 – 50.46)	15.82	(1.62 – 154.99)	25.17	(2.63 – 240.60)
High TG							
Model 1	1.00	1.95	(0.60 – 6.33)	1.45	(0.44 – 4.75)	2.13	(0.65 – 6.99)
Model 2	1.00	2.15	(0.63 – 7.34)	1.45	(0.43 – 4.89)	2.42	(0.70 – 8.34)
High BP							
Model 1	1.00	1.56	(0.48 – 5.09)	0.67	(0.20 – 2.27)	4.07	(1.12 – 14.76)
Model 2	1.00	2.10	(0.57 – 7.70)	0.52	(0.14 – 1.93)	5.86	(1.41 – 24.38)
High blood glucose							
Model 1	1.00	3.09	(0.61 – 15.72)	4.13	(0.85 – 20.13)	2.53	(0.47 – 13.56)
Model 2	1.00	3.35	(0.62 – 17.99)	3.16	(0.64 – 15.61)	2.41	(0.43 – 13.47)

Model 1: adjusted for age; Model 2: Model 1 plus additional adjustments for smoking, alcohol consumption, and physical exercise.

Table S2. Odds ratios (OR) and 95% confidence intervals (CI) for metabolic syndrome and associated risk factors according to the LPS/HDL ratio in women.

	LPS/HDL quartiles						
	Quartile 1 (<0.7)	Quartile 2 (0.7–1.2)		Quartile 3 (1.2–2.1)		Quartile 4 (≥2.1)	
	OR	OR	(95% CI)	OR	(95% CI)	OR	(95% CI)
Metabolic syndrome							
Model 1	1.00	1.32	(0.30 – 5.81)	1.04	(0.22 – 4.88)	0.80	(0.17 – 3.76)
Model 2	1.00	1.28	(0.28 – 5.81)	1.07	(0.22 – 5.06)	0.86	(0.18 – 4.13)
High waist circumference							
Model 1	1.00	1.31	(0.31 – 5.58)	1.01	(0.22 – 4.59)	0.68	(0.13 – 3.44)
Model 2	1.00	1.28	(0.29 – 5.56)	1.03	(0.23 – 4.65)	0.69	(0.13 – 3.56)
Low HDL cholesterol							
Model 1	1.00	1.00	(0.29 – 3.46)	2.10	(0.64 – 6.86)	4.60	(1.38 – 15.26)
Model 2	1.00	0.93	(0.26 – 3.30)	2.07	(0.63 – 6.78)	4.33	(1.29 – 14.56)
High TG							
Model 1	1.00	1.32	(0.30 – 5.85)	1.39	(0.31 – 6.18)	2.01	(0.50 – 8.09)
Model 2	1.00	1.27	(0.28 – 5.73)	1.36	(0.31 – 6.09)	1.86	(0.45 – 7.64)
High BP							
Model 1	1.00	2.12	(0.51 – 8.80)	1.39	(0.31 – 6.20)	3.41	(0.87 – 13.32)
Model 2	1.00	1.90	(0.43 – 8.36)	1.34	(0.29 – 6.12)	2.88	(0.71 – 11.70)
High blood glucose							
Model 1	1.00	4.66	(0.48 – 45.62)	2.18	(0.18 – 25.97)	2.79	(0.27 – 29.25)
Model 2	1.00	4.55	(0.44 – 47.26)	2.36	(0.19 – 29.13)	3.48	(0.31 – 38.58)

Model 1: adjusted for age; Model 2: Model 1 plus additional adjustments for alcohol consumption and physical exercise; BP, blood pressure; TG, triglyceride.

Table S3. The association between the LPS/HDL ratio and metabolic syndrome based on blood Cd concentrations.

	Men		Women	
	**OR**	**(95% CI)**	**OR**	**(95% CI)**
Metabolic syndrome				
Cd < 50th percentile	1.29	(0.58 – 2.86)	1.51	(0.61 – 3.73)
Cd ≥ 50th percentile	3.07	(1.41 – 6.68)	0.73	(0.35 – 1.51)
High waist circumference				
Cd < 50th percentile	1.67	(0.76 – 3.64)	1.81	(0.76 – 4.27)
Cd ≥ 50th percentile	1.56	(0.86 – 2.83)	0.53	(0.22 – 1.30)
Low HDL cholesterol				
Cd < 50th percentile	1.38	(0.57 – 3.29)	2.21	(1.12 – 4.39)
Cd ≥ 50th percentile	3.50	(1.33 – 9.22)	1.57	(0.84 – 2.91)
High TG				
Cd < 50th percentile	1.15	(0.58 – 2.25)	1.33	(0.59 – 3.00)
Cd ≥ 50th percentile	1.60	(0.89 – 2.89)	1.30	(0.69 – 2.45)
High BP				
Cd < 50th percentile	1.90	(0.88 – 4.08)	1.79	(0.84 – 3.80)
Cd ≥ 50th percentile	1.41	(0.74 – 2.70)	1.17	(0.62 – 2.19)
High blood glucose				
Cd < 50th percentile	1.14	(0.47 – 2.74)	1.99	(0.59 – 6.67)
Cd ≥ 50th percentile	1.52	(0.74 – 3.14)	1.16	(0.46 – 2.93)

Model adjusted for age, smoking, alcohol consumption and physical exercise in men, age, alcohol consumption and physical exercise in women. Cd, Cadmium; BP, blood pressure; TG, triglyceride. The cutoff values for 50th percentile of Cd are 0.96 μg/L for men, 1.28 μg/L for women.

© 2015 by the authors; licensee MDPI, Basel, Switzerland. This article is an open access article distributed under the terms and conditions of the Creative Commons Attribution license (http://creativecommons.org/licenses/by/4.0/).
